# Transcriptomics Modeling of the Late-Gestation Fetal Pituitary Response to Transient Hypoxia

**DOI:** 10.1371/journal.pone.0148465

**Published:** 2016-02-09

**Authors:** Charles E. Wood, Eileen I. Chang, Elaine M. Richards, Maria Belen Rabaglino, Maureen Keller-Wood

**Affiliations:** 1 Department of Physiology and Functional Genomics, University of Florida College of Medicine, Gainesville, Florida 32610, United States of America; 2 Department of Pharmacodynamics, University of Florida College of Pharmacy, Gainesville, Florida 32610, United States of America; 3 Department of Physiology and Functional Genomics, University of Florida College of Medicine, Gainesville, Florida 32610, United States of America; University of Southampton, UNITED KINGDOM

## Abstract

**Background:**

The late-gestation fetal sheep responds to hypoxia with physiological, neuroendocrine, and cellular responses that aid in fetal survival. The response of the fetus to hypoxia represents a coordinated effort to maximize oxygen transfer from the mother and minimize wasteful oxygen consumption by the fetus. While there have been many studies aimed at investigating the coordinated physiological and endocrine responses to hypoxia, and while immunohistochemical or *in situ* hybridization studies have revealed pathways supporting the endocrine function of the pituitary, there is little known about the coordinated cellular response of the pituitary to the hypoxia.

**Results:**

Thirty min hypoxia (from 17.0±1.7 to 8.0±0.8 mm Hg, followed by 30 min normoxia) upregulated 595 and downregulated 790 genes in fetal pituitary (123–132 days’ gestation; term = 147 days). Network inference of up- and down- regulated genes revealed a high degree of functional relatedness amongst the gene sets. Gene ontology analysis revealed upregulation of cellular metabolic processes (e.g., RNA synthesis, response to estrogens) and downregulation of protein phosphorylation, protein metabolism, and mitosis. Genes found to be at the center of the network of upregulated genes included genes important for purine binding and signaling. At the center of the downregulated network were genes involved in mRNA processing, DNA repair, sumoylation, and vesicular trafficking. Transcription factor analysis revealed that both up- and down-regulated gene sets are enriched for control by several transcription factors (e.g., SP1, MAZ, LEF1, NRF1, ELK1, NFAT, E12, PAX4) but not for HIF-1, which is known to be an important controller of genomic responses to hypoxia.

**Conclusions:**

The multiple analytical approaches used in this study suggests that the acute response to 30 min of transient hypoxia in the late-gestation fetus results in reduced cellular metabolism and a pattern of gene expression that is consistent with cellular oxygen and ATP starvation. In this early time point, we see a vigorous gene response. But, like the hypothalamus, the transcriptomic response is not consistent with mediation by HIF-1. If HIF-1 is a significant controller of gene expression in the fetal pituitary after hypoxia, it must be at a later time.

## Introduction

The late-gestation fetal sheep responds to hypoxia with physiological, neuroendocrine, and cellular responses that aid in fetal survival [1–4]. The fetal pituitary also plays a critical role in the initiation of parturition in this and ruminant species [5, 6]. The response of the fetus to hypoxia represents a coordinated effort to maximize oxygen transfer from the mother and minimize wasteful oxygen consumption by the fetus. The cardiovascular response to fetal hypoxia features a redistribution of fetal combined ventricular output with increased blood flow to the brain and pituitary [7]. Although this response minimizes the loss of oxygen delivery to the tissue, it is unlikely to prevent cellular oxygen deprivation. While there have been many studies aimed at investigating the coordinated physiological and endocrine responses to hypoxia, and while immunohistochemical or *in situ* hybridization studies have revealed pathways supporting the endocrine function of the pituitary [8–11], there is little known about the coordinated cellular response of the pituitary to the hypoxia.

In the present study, we assess pituitary responses to hypoxia using a systems biology-based analysis of the transcriptomics responses. We propose that systems modeling can be used to detect the cellular response to hypoxia and that this modeling methodology will reveal cellular oxygen deprivation as expected based on the physiological data. We have successfully used this approach to identify hypothalamic responses to hypoxia in late-gestation fetal sheep, using systems analysis to identify important cellular responses that are not biased by focus on one group of neurons or cellular phenotype. We reported, for example, that few genes accounting for the hypothalamic transcriptomics response to hypoxia were transcriptionally controlled by HIF-1 [3].

## Results

Infusion of nitrogen into the maternal trachea decreased fetal P_a_O_2_ from 17.0±1.7 to 8.0±0.8 mm Hg. Maternal hyperventilation in response to the hypoxia decreased fetal P_a_CO_2_ from 50.3±1.9 to 46.8±1.9 mm Hg and increased fetal pH_a_ from 7.40±0.01 to 7.42±0.01. In the normoxic fetuses, P_a_O_2_, P_a_CO_2_, and pH_a_ were 18.7±1.8 mm Hg, 51.7±0.7 mm Hg, and 7.37±0.01, respectively.

**Fig 1:**
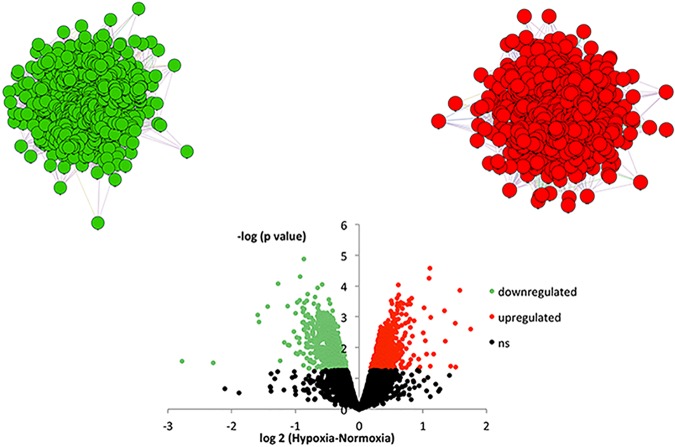
Volcano plot illustrating increases and decreases in pituitary gene expression in hypoxic versus normoxic fetuses. Differences between groups are represented on the x-axis as differences in expressed in values of logarithm of 2. Probability values are represented on the y-axis as values of logarithm of 10. Points drawn above the dashed line are statistically significant (*P*<0.05). Statistically significant increases in gene expression are shown in red and statistically significant decreases in gene expression are shown in green. Above the volcano plot are inferred networks of the upregulated (red) and downregulated (green) genes, plotted as force-directed layouts of the networks.

Hypoxia upregulated 595 genes and downregulated 790 genes in the pituitary (Fig 1). Both the upregulated and downregulated genes could be organized into single networks (Fig 1: downregulated–green; upregulated–red). The parameters of the inferred networks are reported in Table 1. Interestingly, nearly all of the differentially regulated genes could be organized into networks (n = 576 upregulated and n = 790 downregulated genes) when genes were connected by functional parameters, including genetic and physical interactions or if genes belonged to the same pathway or shared protein domain (Table 1). Furthermore, inference of the networks on the basis of “genetic interaction” alone also included most of the upregulated (n = 520) and downregulated (n = 694) genes (Table 1).

**Table 1:** Parameters of inferred networks of up- and down-regulated genes.

Number of Inference Parameters	7 Parameters		5 Parameters		1 Parameter	
Network Inference	Upregulated Genes	Downregulated Genes	Upregulated Genes	Downregulated Genes	Upregulated Genes	Downregulated Genes
# Nodes in Network	595	790	576	790	520	694
# Edges in Network	57772	103246	8314	16333	6992	13510
Co-Expression (% edges)	56.50	47.11	excluded	excluded	excluded	excluded
Physical Interactions (% edges)	26.12	44.09	78.15	85.59	excluded	excluded
Co-Localization (% edges)	10.29	5.84	excluded	excluded	excluded	excluded
Genetic Interactions (% edges)	5.27	1.72	15.8	11.48	100	100
Pathway (% edges)	1.38	0.85	4.38	2.00	excluded	excluded
Predicted (% edges)	0.24	0.22	0.87	0.55	excluded	excluded
Shared Protein Domains (% edges)	0.21	0.16	0.80	0.39	excluded	excluded

**Fig 2:**
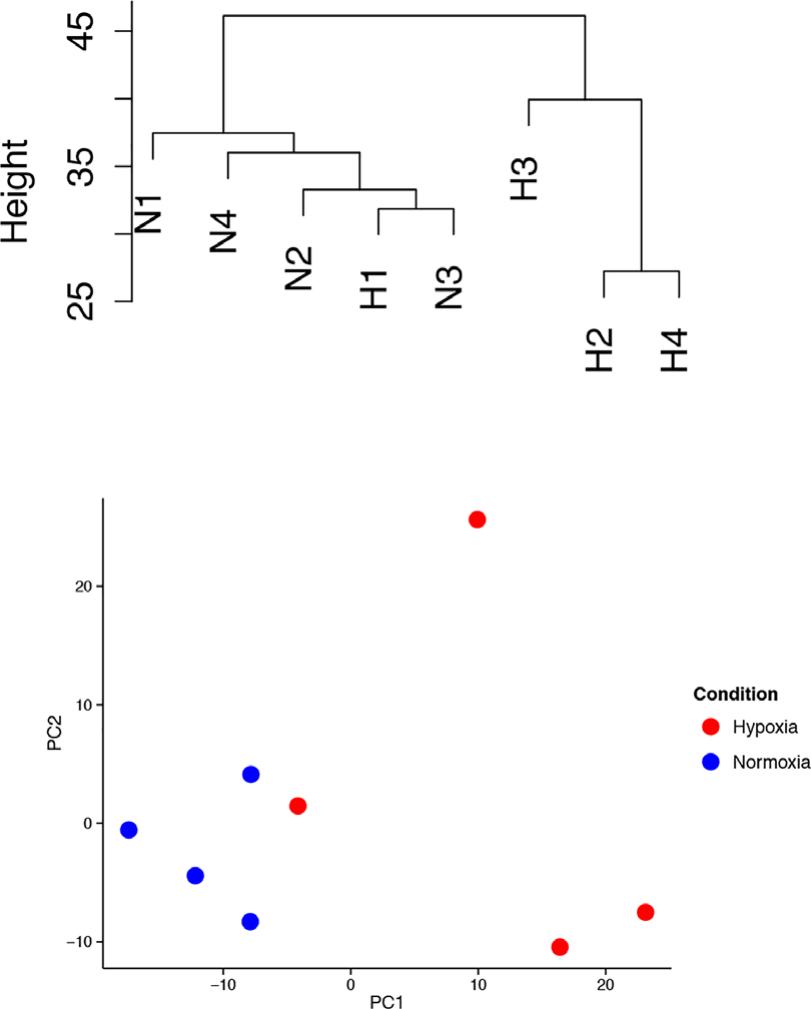
Hierarchical clustering. Top panel: Hierarchical clustering of gene expression in the normoxic (N1, N2, N3, and N4) and hypoxic (H1, H2, H3, H4) fetal pituitaries. Bottom panel: Principal component analysis of gene expression in normoxic (red symbols) and hypoxic (blue symbols) fetal pituitaries.

Hierarchical clustering of gene expression in the arrays revealed that, in general, the hypoxic pituitaries grouped separate from the normoxic pituitaries (Fig 2, top). One exception to this is that one of the hypoxia pituitaries was more closely related to the normoxic pituitaries. Principal component analysis corroborated the clustering analysis, showing that both groups are separated along the X-axis or the first principal component, which account for most variability between samples (Fig 2, bottom panel).

**Fig 3:**
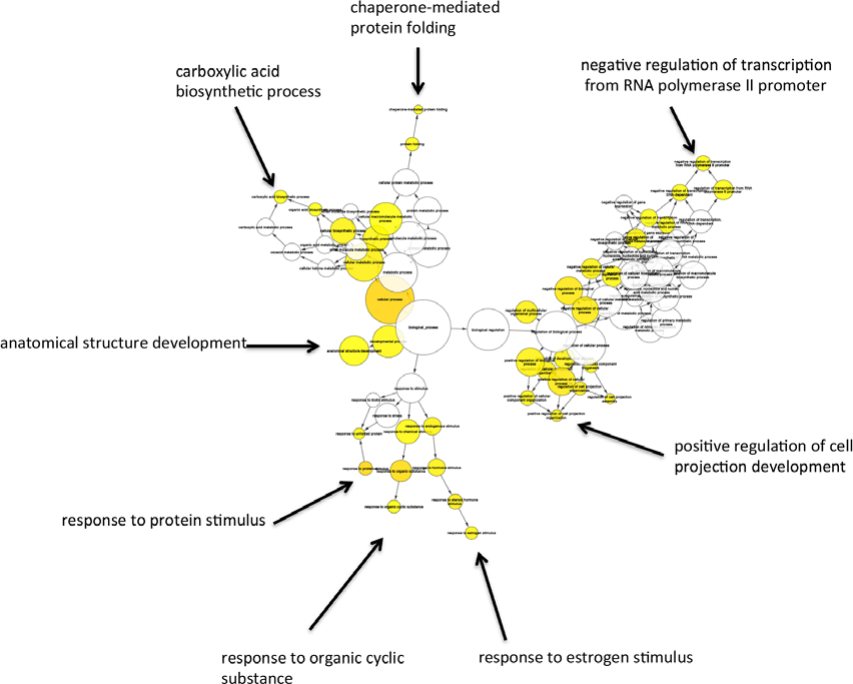
Network of gene ontology terms significantly (*P*<0.05) associated with the genes that were upregulated after hypoxia. Note that the highest order ontology terms (e.g., glucocorticoid signaling, or prostaglandin biosynthesis, etc) are in the outermost region of the network, while more basic ontology terms (e.g., “biological process”, which is not clearly shown in this figure because of reduced size of the node labels) are in the center of the network. The intensity of the color of each node denotes statistical significance (*i*.*e*., orange greater statistical significance than yellow, and uncolored nodes are not statistically significant). All nodes labeled with arrows are statistically significant.

**Fig 4:**
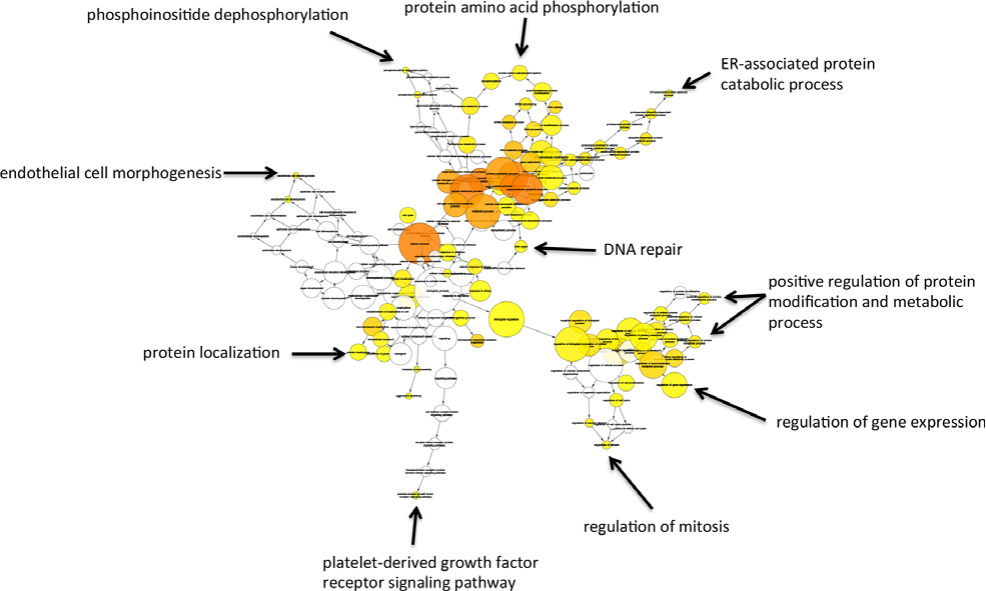
Network of gene ontology terms significantly (*P*<0.05) associated with the genes that were downregulated after hypoxia. The intensity of the color of each node denotes statistical significance (*i*.*e*., orange greater statistical significance than yellow, and uncolored nodes are not statistically significant). All nodes labeled with arrows are statistically significant.

The inferred networks of Gene Ontology (GO) terms for up- and down-regulated genes are reported in Figs 3 and 4, respectively. GO analysis of these differentially regulated genes revealed statistically significant upregulation (n = 214 genes, *P* = 0.01) of cellular metabolic processes, including regulation of transcription from RNA polymerase II promoter (n = 41, *P*<0.05), positive regulation of cell projection organization (n = 16, *P*<0.02), response to estrogen stimulus (n = 13, *P*<0.02), response to organic cyclic substance (n = 14, *P*<0.05), response to protein stimulus (n = 17, *P*<0.001), anatomical structure development (n = 121, *P*<0.05), carboxylic acid biosynthetic process (n = 15, *P*<0.05), and chaperone-mediated protein folding (n = 4, *P* < .0005). GO analysis of downregulated genes revealed changes in protein amino acid phosphorylation (n = 50, *P*<0.02), ER-associated protein catabolic process (n = 7, *P*<0.02), DNA repair (n = 27, *P*<0.05), positive regulation of protein modification (n = 24, *P*<0.02), positive regulation of protein metabolic process (n = 28, *P*<0.02), regulation of gene expression (n = 168, *P*<0.05), regulation of mitosis (n = 10, *P*<0.02), platelet-derived growth factor receptor signaling pathway (n = 5, *P*<0.05), protein localization (n = 62, *P*<0.05), endothelial cell morphogenesis (n = 3, *P*<0.02), and phosphoinositide dephosphorylation (n = 3, *P*<0.05).

Analysis of the most highly connected nodes within the networks (highest values of calculated stress by CentiScaPe in the networks inferred using the 7 parameters) revealed the genes most likely to be central to the functional response. From each network, the 10 genes with the highest calculated value of “stress” are reported in Tables 2 and 3.

**Table 2:** Top ten most highly-related nodes in network of upregulated genes.

Ensemble Gene Name	CentiScaPe Stress	CentiScaPe Radiality	
ACTR3	85670	3.297800338	ARP3 actin-related protein 3 homolog (yeast)
ATIC	51024	3.221658206	5-aminoimidazole-4-carboxamide ribonucleotide formyltransferase/IMP cyclohydrolase
SET	47252	3.241962775	SET nuclear oncogene
GTF2E1	41934	3.194585448	general transcription factor IIE, polypeptide 1, alpha 56kDa
TBL1XR1	41020	3.223350254	transducin (beta)-like 1 X-linked receptor 1
BLVRB	40264	3.184433164	biliverdin reductase B (flavin reductase (NADPH))
ISL1	39454	3.186125212	ISL LIM homeobox 1
COMMD1	38864	3.16751269	copper metabolism (Murr1) domain containing 1
YARS	38204	3.21319797	tyrosyl-tRNA synthetase
FOXP1	37740	3.230118443	forkhead box P1

**Table 3:** Top ten most highly related nodes in network of downregulated genes.

Ensemble Gene Name	CentiScaPe Stress	CentiScaPe Radiality	
SEC24B	130152	3.338010204	SEC24 family, member B (S. cerevisiae)
CAST	136268	3.322704082	calpastatin
HNRNPA2B1	87230	3.255102041	heterogeneous nuclear ribonucleoprotein A2/B1
KCTD12	119906	3.265306122	potassium channel tetramerisation domain containing 12
EPS15	84846	3.253826531	epidermal growth factor receptor pathway substrate 15
ANKRD17	77510	3.253826531	ankyrin repeat domain 17
UBA2	63736	3.242346939	ubiquitin-like modifier activating enzyme 2
REV1	65578	3.241071429	REV1 homolog (S. cerevisiae)
SRSF11	63348	3.229591837	serine/arginine-rich splicing factor 11
LRBA	63760	3.233418367	LPS-responsive vesicle trafficking, beach and anchor containing

**Fig 5:**
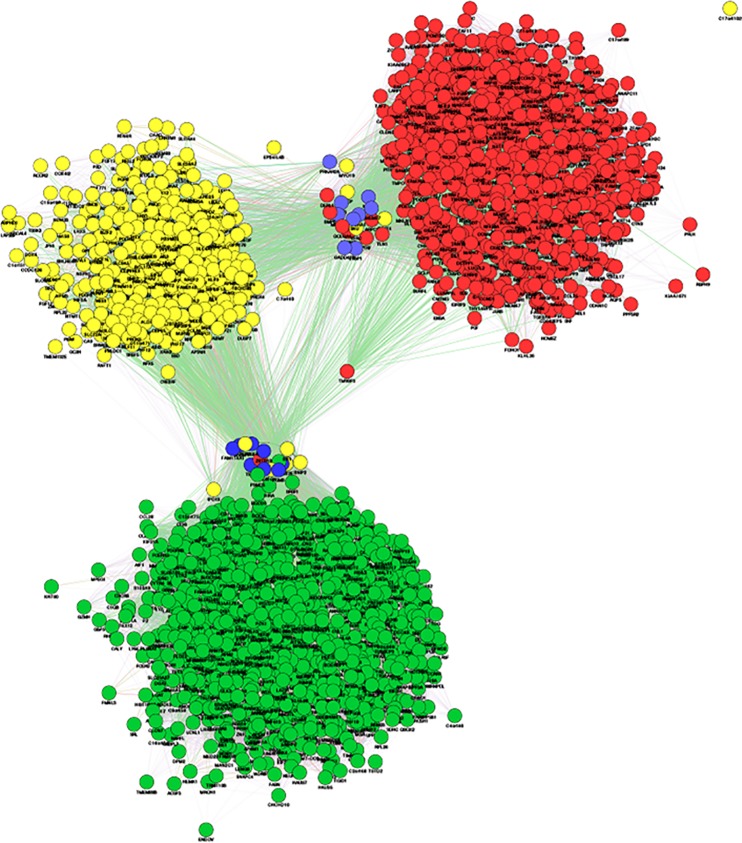
Overlap of inferred networks of differentially-regulated genes and genes that are potentially controlled by HIF-1. Genes that were significantly upregulated in the pituitary after hypoxia are represented as a network with red nodes (as in Fig 1). Genes that were significantly downregulated in the pituitary after hypoxia are represented as a network with green nodes (as in Fig 1). Genes with HIF-1 consensus binding sites in their putative promoter regions (-2 to +2 kB) are represented as yellow nodes. Genes that were found to be in both the red- and yellow networks, and genes that were found to be in both the green- and yellow- networks after network merge are colored blue. The merged network is represented in a force-directed layout.

Results of transcription factor (TF) analysis (WebGestalt) for top ten TF’s significantly associated with up- or down-regulated genes are reported in Tables 4 and 5. TF’s significantly associated with *both* the up- and down-regulated gene sets contained SP1 (152 upregulated and 145 downregulated genes), MAZ (100 upregulated and 108 downregulated genes), LEF1 (83 upregulated and 103 downregulated genes), NRF1 (58 upregulated and 70 downregulated genes), ELK1 (61 upregulated and 78 downregulated genes), NFAT (71 upregulated and 92 downregulated genes), E12 (85 upregulated and 110 downregulated genes), PAX4 (57 upregulated and 70 downregulated genes), MYC (51 upregulated and 59 downregulated), ETS2 (44 upregulated and 64 downregulated), FOXO4 (67 upregulated and 113 downregulated). E4F1 (n = 41) was associated only with upregulated genes. Fig 5 illustrates the small overlap of the differentially regulated genes with genes whose promoter regions contain HIF-1 binding sites. Only 15 upregulated and 14 downregulated genes contained consensus HIF-1 binding regions in the promoter regions (Broad Institute, V$HIF1_Q3 and V$HIF1_Q5).

**Table 4:** Top 10 transcription factor binding sites significantly over-represented in the network of genes that were significantly upregulated in response to transient hypoxia in fetal pituitary.

Transcription Factor Binding Site	# Genes	# Occurrences	Adjusted Probability
hsa_GGGCGGR_V$SP1_Q6	159	2891	1.08E-49
hsa_GGGAGGRR_V$MAZ_Q6	104	2250	8.25E-25
hsa_CTTTGT_V$LEF1_Q2	91	1939	5.34E-22
hsa_CACGTG_V$MYC_Q2	62	1015	3.70E-20
hsa_RCGCANGCGY_V$NRF1_Q6	58	894	3.96E-20
hsa_SCGGAAGY_V$ELK1_O2	66	1176	9.48E-20
hsa_GTGACGY_V$E4F1_Q6	46	646	1.83E-17
hsa_TGGAAA_V$NFAT_Q4_01	76	1871	5.24E-15
hsa_CAGGTG_V$E12_Q6	85	2450	5.46E-13
hsa_GGGTGGRR_V$PAX4_O3	57	1278	8.97E-13

**Table 5:** Top 10 transcription factor binding sites significantly over-represented in the network of genes that were significantly downregulated in response to transient hypoxia in fetal pituitary.

Transcription Factor Binding Site	# Genes	# Occurrences	Adjusted Probability
hsa_GGGCGGR_V$SP1_Q6	153	2891	1.64E-28
hsa_CTTTGT_V$LEF1_Q2	121	1939	1.77E-28
hsa_TTGTTT_V$FOXO4_O1	120	2037	3.91E-26
hsa_RCGCANGCGY_V$NRF1_Q6	72	894	8.30E-23
hsa_SCGGAAGY_V$ELK1_O2	80	1176	7.34E-21
hsa_GGGAGGRR_V$MAZ_Q6	116	2250	1.03E-20
hsa_TGGAAA_V$NFAT_Q4_O1	99	1871	2.86E-18
hsa_CAGGTG_V$E12_Q6	115	2450	1.87E-17
hsa_GCCATNTTG_V$YY1_Q6	43	419	2.15E-17
hsa_GGGTGGRR_V$PAX4_O3	70	1278	1.62E-13

We performed targeted qPCR on several genes of interest, reported in Table 6. These genes were of interest to us because of what is known about the physiological and endocrine response of the fetal pituitary to hypoxia. We found a significant increase in FSH mRNA abundance, but no statistically significant change in gene expression for POMC, PRL, or LH. Estrogen receptor alpha (ESR1) and the g protein-coupled membrane estrogen receptor (GPER) were not altered by hypoxia, but estrogen receptor beta (ESR2) was significantly decreased. Of three measured isoforms of 17β-hydroxysteroid dehydrogenase (interconverts estradiol and estrone), only HSD17B4 was significantly changed (increased).

**Table 6:** qPCR estimates (dCt±SEM) of mRNA abundance of genes of interest and comparison to expression on microarray.

Target	qPCR nmx (- ΔΔCt±SEM)	qPCR hypx (- ΔΔCt±SEM)	Array nmx Δlog_2_(intensity)	Array hypx Δlog_2_(intensity)
ESR1	0.0±0.2	-0.6±0.4	0.0±0.1	0.2±0.1
ESR2	0.0±0.2	-0.8±0.3*	0.0±0.1	-0.1±0.1
GPER	0.0±0.3	0.4±0.3	ND	ND
NOS2	0.0±0.2	-0.4±0.3	0.0±0.2	0.1±0.2
LH	0.0±0.1	0.7±0.7	0.0±0.1	0.3±0.2
FSH	0.0±0.3	1.0±0.2**	0.0±0.2	0.4±0.1
PRL	0.0±0.4	0.1±1.1	0.0±0.01	0.0±0.03
POMC	0.0±1.7	-0.9±1.7	0.0±0.1	0.0±0.1
PTGS1	0.0±0.1	-0.2±0.1	0.0±0.1	-0.3±0.1
PTGS2	ND	ND	0.0±0.1	-0.9±0.2*
HSD17B2	0±0.5	0.8±0.3	ND	ND
HSD17B4	0.0±0.1	0.6±0.1*	ND	ND
HSD17B10	0±0.1	-0.4±0.3	ND	ND
CXCL14	0.0±0.4	1.4±0.4*	0.0±0.6	1.2±0.7*
CAPN3	0.0±0.3	-1.5±0.1**	0.0±0.3	-1.6±0.3*
MSTN	0.0±0.3	-0.9±0.3*	0.0±0.3	-1.0±0.2*
CATHL5	0.0±0.7	-0.3±0.5	0.0±0.7	-2.3±0.9*
CHST15	0.0±0.3	0.5±0.2	0.0±0.5	1.1±0.4*
TAC1	0.0±0.5	4.4±0.6***	0.0±0.4	1.5±0.7*

* *P*<0.05

** *P*<0.01

*** *P*<0.001.

Six differentially-expressed genes on the array (CXCL14, CAPN3, MSTN, CATHL5, CHST15, TAC1) were also analyzed by qPCR (Table 6). The changes in the array for 4 of these genes were reflected in statistically significant changes as measured by qPCR. The measured change in the other 2 genes (CATHL5, CHST15) by qPCR were not statistically significant, but there was a trend in the same direction as in the array.

## Discussion

The fetus lives and develops in an environment that is hypoxic relative to the adult. The partial pressure of oxygen in the arterial blood of the fetal sheep is normally approximately 18–22 mm Hg, while the partial pressure of oxygen in the arterial blood of the adult is closer to 100 mm Hg [12–14]. While the fetus appears to live “at the top of Mount Everest” [15], there are important physiological adaptations that maximize the delivery of oxygen to the developing, physiologically active tissues. Fetal erythrocytes contain fetal hemoglobin, which binds oxygen more tightly that adult hemoglobin [16], and fetal cardiac output (hence blood flow to the systemic tissues and brain) is approximately 2–3 times that of the adult, normalized to body weight [17]. Notwithstanding these adaptations to life in the uterus, disruptions in oxygen delivery to the fetus can be damaging to the developing tissues. Chronic hypoxia, for example, can cause intrauterine growth restriction [18]. Acute hypoxia (transient hypoxia, or hypoxia and reoxygenation), which can result from, for example, exposure to high altitude or transient interruption of oxygen delivery during umbilical cord occlusion can also be damaging to the fetus. Transient hypoxia stimulates physiological responses that feature redistribution of fetal cardiac output and partially spare the oxygen delivery to brain and heart [19]. Interestingly, while we understand aspects of the physiological response to transient hypoxia and reoxygenation, we do not understand the cellular responses of many of the tissues–including pituitary—that are most important for survival and well-being of the fetus. Of note is that, in the present experiments, the normoxia group had P_a_O_2_ values that were slightly low compared to what we consider normoxic conditions (mean P_a_O_2_ = 17 mm Hg). We believe that this was the result of the choice to use twin pregnancies in this study. Twin fetuses have been reported to have a nonsignificant tendency to lower P_a_O_2_ values than singleton fetuses [20]. This is in general agreement with another report of a statistically significant effect of fetal number on P_a_O_2_ [21].

The pituitary is integral to the fetal endocrine stress response to hypoxia. Hypoxia stimulates an increase in fetal adrenocorticotropin (ACTH) [22] and vasopressin [23] secretion. ACTH, in turn, stimulates adrenal secretion of cortisol [24] which has many downstream actions including negative feedback inhibition of fetal ACTH secretion [25]. Vasopressin aids in the cardiovascular response to hypoxia, stimulating peripheral vasoconstriction with the resultant redistribution of combined ventricular output from peripheral vasculature to the fetal heart and brain [26]. The pituitary is likely to have a high metabolic rate (oxygen consumption rate) because of its endocrine activity. Perhaps not surprisingly, the oxygen consumption rate of the fetal pituitary has not been measured. However, pituitary blood flow has been measured, allowing an estimation of changes in oxygen delivery during acute hypoxia. In chronically catheterized fetal sheep, Richardson and colleagues reported that blood flow to the pituitary increased only about 19% (115 to 137 mL/min/100 gram tissue wet weight) when blood oxygen partial pressures were reduced from approximately 25 to approximately 18 mm Hg [7]. A reduction in the partial pressure of oxygen by 50% in the fetus reduces oxygen content by more than 50%, owing to the steep slope of the fetal oxygen-hemoglobin dissociation curve at fetal arterial oxygen tensions [27]. This situation is exacerbated by the fact that the blood perfusing the pituitary gland is both arterial and from the hypothalamo-hypophyseal portal vasculature [28]. Based on this understanding of oxygenation and blood flow in the fetus, we believe that acute hypoxia as produced in the present study causes marked cellular hypoxia in the pituitary.

The pituitary is a heterogeneous tissue, containing the endocrine secreting cells of the anterior pituitary, and the neurons and pituicytes (glia) of the posterior pituitary. The acute (1 hour) response of the pituitary to hypoxia is characterized by changes in transcription of genes controlling metabolism, RNA synthesis and splicing, protein synthesis and modification, and alteration in tissue immune activity. Interestingly, this is reminiscent of the response of the fetal hypothalamus to the same degree of hypoxia (acute, 1 hour post-hypoxia) [3]. Also reminiscent of the hypothalamic response to hypoxia is the apparently minimal involvement of HIF-1 in the response. Nevertheless, we cannot explain the commonality of the responses based on known pathways. Comparing genes upregulated by hypoxia in hypothalamus versus pituitary, we find only 45 genes that are commonly upregulated and 120 genes that are commonly downregulated (approximately 8% and 13%, respectively, in each tissue). Nevertheless, there is a substantial overlap of GO terms between hypothalamus and pituitary, perhaps suggesting that among the responses to hypoxia are a “core” of cellular responses that are shared by these two tissues. Commonly downregulated in both tissues are genes controlling mitotic anaphase, and DNA methylation. Commonly upregulated are genes associated with chaperone mediated protein folding, BMP signaling, regulation transcription from RNA polymerase II promoter, binding of sequence-specific DNA binding transcription factor activity.

The apparently minimal involvement of HIF-1 (Hypoxia Inducible Factor-1) as a mediator of the pituitary responses to hypoxia is suggested by the small number of differentially regulated genes whose promoter regions contain consensus HIF-1 binding sites (Fig 5). While 29 of the differentially expressed genes do contain HIF-1 binding sites, the functionality of HIF in the response of these genes to hypoxia in various tissues (and with varied lengths of exposure to hypoxia) is unknown at present. In the interpretation of the present results, it is important to remember that this is a single time point (1 hour after onset and 0.5 hours after cessation of hypoxia). It is possible that, at later time points, HIF-1 might be an important regulator of the genomic response. Nevertheless, the apparent minimal dependency on HIF-1 at one hour suggests that the acute response to the hypoxia is perhaps more likely to involve cellular responses to declining energetics, reminiscent of the mechanism of response to hypoxia in vascular smooth muscle cells by Koltsova and colleagues [29]. This suggestion is supported by the genes that are most central within the network of upregulated genes. ACTR3, the gene at the center of the network, is the ATP binding protein component of the Arp2/3 complex which, in turn, is involved in maintenance of cell shape and motility [30]. ATIC, the gene that is next most central to the upregulated network, is involved in purine biosynthesis [31]. Interestingly, COMMD1 is known to inhibit HIF-1-stimulated gene transcription [32] and is also among the 10 most centrally-located genes in the upregulated network. Genes most centrally-located within the downregulated network include genes involved in mRNA processing (HNRNPA2B1, SRSF11), DNA repair (REV1), sumoylation (UBA2), and vesicular trafficking (SEC24B). Also among the genes central to the downregulated network is KCTD12, which is involved in K^+^ channel tetramerization [33]. One might expect that, if the primary pituitary cellular response to hypoxia was ATP depletion, there would be downstream effects on membrane K^+^ potential and biosynthesis of K^+^ channel components.

Gene ontology analysis revealed a significant association of upregulated genes with response to steroid hormone stimulus and a significant association of downregulated genes with glucocorticoid receptor signaling pathway. Association of both up- and down-regulated genes with steroid signaling makes sense relative to what we know about the fetal pituitary response to hypoxia. Importantly, we know that the fetal sheep increases the activity of the hypothalamus-pituitary-adrenal axis during hypoxia, with substantial increases in fetal plasma ACTH and cortisol. The glucocorticoid receptor (NR3C1) is downregulated in the present study, likely the result of receptor downregulation after ligand binding [34]. Interestingly, POMC mRNA was not significantly altered on the array or in qPCR assay (Table 4). The lack of change of POMC mRNA suggests that the expected upregulation (in response to increased POMC/ACTH secretion) must occur later. In addition to glucocorticoid signaling, we expect estrogen signaling be altered after hypoxia. While neither ESR1 nor ESR2 (ERα and ERβ, respectively) were significantly altered on the array, we measured both mRNA’s using qPCR because we had previously reported that cerebral hypoperfusion (caused by brachiocephalic occlusion) increased ERα mRNA [35]. In the present study, hypoxia increased ESR2 (ERβ) mRNA significantly, with an apparent increase in mean ESR1 expression level that was not statistically significant (Table 4). We previously reported that brachiocephalic occlusion increased LH, but not FSH or PRL mRNA [36]. In the present experiment, we found that hypoxia increased FSH, but not LH or PRL mRNA.

While the transcriptomics modeling appears to reveal a picture of ATP depletion causing downstream effects on gene expression, there are several limitations to this study that deserve comment. First, this study addresses a single time point of 1 hour, designed to find the early transcriptomics responses to the oxygen deprivation. While we call this an “acute” response, it recognized that some investigators might interpret “acute” to include all time points up to 4 hours post stimulus. Gene expression at this time could also be denoted “immediate-early” gene expression [37]. Following from this concept, it is important to state that the transcriptomics response to hypoxia at later time points will be different from that measured in the present study, and indeed perhaps directed by it. Second, the conclusion that the transcriptomics modeling does not implicate HIF1 in the response is tempered by the reminder that this is a report of transcriptomics modeling that takes into account downstream gene transcription controls, but does not address the control of the actual protein components of HIF1α, which are influenced by both transcriptional and post-translational control mechanisms. Third, the database accessed by Webgestalt for these analyses only considers transcription factor binding sites within 2 kb of the transcription start site, so may not capture genes controlled from transcription factor binding sites more distant from this area.

The multiple analytical approaches used in this study suggests that the acute response to 30 min of transient hypoxia in the late-gestation fetus results in reduced cellular metabolism and a pattern of gene expression that is consistent with cellular oxygen and ATP starvation. In this early time point, we see a vigorous gene response. But, like the hypothalamus, the transcriptomic response is not consistent with mediation by HIF-1. If HIF-1 is a significant controller of gene expression in the fetal pituitary after hypoxia, it must be at a later time. Consistent with responses in the hypothalamus, we have also found that the pituitary response to hypoxia includes estrogen-sensitive pathways. While the stimulation of estrogen-sensitive pathways is not likely to involve acute increases in circulating plasma estradiol concentrations, the present results suggest that there are hypoxia-induced changes in estrogen receptor abundance and changes in at least some of the enzymes that interconvert estradiol and estrone.

## Materials and Methods

### Experimental animals

Time-dated twin fetal sheep were chronically catheterized between 123 and 132 days’ gestation (term = 147 days) as previously described [38]. One fetus in each pregnancy is included in this analysis. Each fetus was prepared with femoral arterial and venous catheters and one additional catheter for access to amniotic fluid. The pregnant ewe was prepared with femoral arterial and vein catheters and one additional catheter placed into the maternal trachea for administration of nitrogen gas [35, 39]. Each animal was fully recovered from surgery at the time of study (at least 5 days after surgery). All of the sheep were conscious, freestanding, and exhibiting normal behavior during the experiments. Each fetus was studied only once. All experiments were approved by the University of Florida Institutional Animal Care and Use Committee and were consistent with the Guiding Principles for Experiments Involving Animal and Human Beings [40].

### Experimental Protocol

Two groups of fetuses (n = 4 normoxia and n = 4 hypoxia: 3 male, 1 female in each group) were studied. All experiments were performed while the animals were awake and not exposed to anesthetics. Prior to study, each animal was prepared for study by flushing of catheters, connection of catheters to transducers for measurement of blood pressure, amniotic fluid pressure, and heart rate (data not reported here), and connection of the maternal tracheal catheter to a nitrogen tank for controlled delivery of nitrogen gas. The tracheal catheter did not occlude the maternal trachea or obstruct normal air flow through the trachea to the maternal lungs. Nitrogen was delivered into the maternal trachea in a gentle stream through the tracheal catheter. The dilution of inspired air with nitrogen effectively decreased the content of oxygen in the maternal inspired gas. Owing to the reduction in the fraction of inspired oxygen, the ewe became hypoxic. Because fetal oxygen derives from oxygen in the maternal blood, fetal hypoxia results from maternal hypoxia. Blood samples (5 mL) were drawn before, during (0, 5, 10, 20, and 30 min), and after a 30 min period of hypoxia for measurement of hormonal responses (data not reported here). Additional blood samples (1 mL) were drawn at the same time points for measurement of fetal blood gases. Blood gases were measured using a Radiometer ABL80 blood gas analyzer. Hypoxia was induced by infusion of nitrogen gas into the maternal trachea at a rate sufficient to lower maternal arterial partial pressure of oxygen (P_a_O_2_) by approximately 50%. Because oxygen in the fetus derives from the maternal circulation, a reduction in maternal P_a_O_2_ results in a reduction in fetal P_a_O_2_. Blood gas, blood pressure, and endocrine responses to this stimulus have been recently reported by us [39]. Fetuses in the normoxia group were subjected to the same protocol except that nitrogen was not introduced into the maternal trachea (the tracheal catheter was connected to the valve and nitrogen tank, but the flow of nitrogen was not started). Sixty minutes after the onset of hypoxia or normoxia, the ewe and fetus were euthanized with an overdose of sodium pentobarbital. Tissues were rapidly removed and snap-frozen in liquid nitrogen and stored at -80°C until analyzed. Pituitaries were removed as whole pituitaries: anterior and posterior lobes were not separated. Each pituitary was used in its entirety for mRNA extraction (no protein was extracted).

### mRNA extraction

Messenger RNA was extracted from pituitaries using Trizol (Invitrogen, Carlsbad, CA) as previously described [3, 41], followed by RNeasy PlusMini kits with on-column DNase treatment (Qiazol, Valencia, CA) as previously described [42]. The RNA concentration was determined with a Nanodrop spectrophotometer (ND-1000, ThermoFisher, Wilmington DE) and the integrity of the RNA was measured using an Agilent Bioanalyzer, 2100 model. RNA Integrity Number (RIN) values ranged from 7.6 to 8.7. Five hundred ng of the DNase-treated RNA was labeled with Cyanine 3 (Cy3) CTP with the Agilent Quick Amp kit (5190–0442, New Castle, DE) according to their methodology, purified with the Qiagen RNeasy kit (Valencia, CA) according to Agilent’s revision of the Qiagen protocol as shown in the Quick Amp kit protocol except that the microcentrifugation was performed at room temperature instead of 4°C. The resulting labeled cRNA was analyzed with the NanoDrop spectrophotometer, and the specific activities and the yields of the cRNAs were calculated; these ranged from 10.08 to 17.58 pmol Cy3/μg RNA and from 6.34 to 12.60 μg, respectively. The labeled cRNA was stored at -80°C until use. Microarray hybridization, washing, and scanning were performed as previously described [3, 35, 42].

### Microarray Data Analysis

Raw data were processed with the R software (http://www.r-project.org) employing the limma package to perform background correction and data normalization using the quantile normalization method [43]. Probes that were at least 10% brighter than the negative controls on at least three arrays were retained for further analysis. Similarity between samples according gene expression profile was determined with hierarchical clustering and principal component analysis (ggplot2 package for R) [44]. The processed microarray data were statistically analyzed with the limma package as well, employing moderated t-test that uses empirical Bayes method for small sample size per group (*P*≤0.05). The effect of hypoxia was analyzed by comparing the hypoxic group to normoxic group as the control group. Microarray data have been deposited in the NCBI Gene Expression Omnibus database (GEO accession number GSE69246).

Cytoscape software [45] and its plugin GeneMANIA [46] was used to infer networks of genes that were significantly up- and down-regulated by hypoxia [47–51]. The functional annotation of gene ontogeny for significantly up and down regulated genes were analyzed using the Cytoscape plugin BinGO [52, 53]. Network architecture (node centrality) was analyzed using CentiScaPe [54].

WebGestalt was used for detection of transcription factor binding sites that are statistically significantly over-represented among the differentially regulated genes. This analysis is based on an ORACLE relational database GeneKeyDB, which uses a strong gene and protein centric viewpoint [55].

For analysis of overlap of hypoxia-differentially regulated genes with genes that are potentially regulated by HIF-1, we performed network inference on 331 unique genes that contain consensus binding sites for HIF-1 (GNNKACGTGCGGNN and/or CGTACGTGCNGB; cataloged as V$HIF1_Q3 and V$HIF1_Q5 molecular signature datasets, respectively, in the Gene Set Enrichment Analysis Database of the Broad Institute) [56, 57]. The genes in these molecular signature datasets are known to contain HIF-1 consensus binding sites in the putative promoter regions (-2 to +2 Kb), and are therefore a comprehensive list of genes–as currently known–that are potentially HIF-1 controlled. Overlap of the inferred network with the networks of genes that were significantly up- and down-regulated was performed using the network merge function within Cytoscape.

### Real-time PCR

cDNA made from the same RNA used for the microarray analyses were used for qPCR validation of the microarray findings. The primers were designed based on the known *Ovis aries* and *Bos taurus* genomes for SYBR green or Taqman probe detection in qPCR (Table 7). As the housekeeping control, ovine β-actin primers and probe were used [3]. The RNA expression was normalized by the difference in threshold cycle (ΔCt) between the triplicate mean Ct for each gene and the triplicate mean Ct for β-actin mRNA from the same sample, and these ΔCt values compared between treatment groups. Experimental groups were compared using Student’s unpaired t-test, with the criterion for statistical significance *P*<0.05. Data are presented as mean values±standard error of the mean (SEM).

**Table 7:** Primer and probes used in qPCR analysis.

Gene	Forward Primer	Reverse Primer	Taqman Probe
ESR1	AGGCACACGGGAGCACAT	TTCCATGGGCTTGTAGAAGTCA	CTTCCCTTCCTTCTCACTGTCTCAGCCC
ESR2	GCTCTGGTCTGGGTGATTGC	GTTAGCCAGGCGCATGGA	AAGAGCGGCATGTCCTCCCAGCA
GPER	CTCTTCCTGCAGGTCCAACATGTA	GCGGTCGAAGCTCATCCA	
NOS2	TGATGCAGAAGGCCATGTCA	TCTCCCTGTCTCTGTTGCAAAG	CCCGGGTCAGAGCCACGATCC
LH	CCGCTCCCAGATATCCTCTTC	GTCTGCTGGCTTTGGGAGTTA	TCTAAGGATGCCCCACTTCAATCTCCCA
FSH	CCCAACATCCAGAAAGCATGT	GCACAGCCAGGCACTTTCA	TTCAAGGAGCTGGTGTACGAGACG
PRL	TGAGCTTGATTCTTGGGTTGCT	CCCCGCACCTCTGTGACTA	CTCCTGGAATGACCCTCTGTATCAC
POMC	CCGGCAACTGCGATGAG	GGAAATGGCCCATGACGTACT	AGCCGCTGACTGAGAACCCCCG
PTGS1	GGCACCAACCTCATGTTTGC	TCTTGCCGAAGTTTTGAAGA	TTCTTTGCCCAACACTTCACCCATCA
PTGS2	GCACAAATCTGATGTTTGCATTCT	CTGGTCCTCGTTCATATCTGCTT	TGCCCAGCACTTCACCCATCAATTTT
HSD17B2	CGACAAGAGCTGTCCAAATGG	CTCGTGCCTGAGATGTTTGTTTT	Sybr
HSD17B4	TGGAAGCCCTAAAGCCAGATTAT	ACAAGCCACCATTTTCTTCACA	Sybr
HSD17B10	GTATCCGGGTGATGACCATTG	CTTTATCTGGGAGGGTGGTCAA	Sybr
CXCL14	ACGGGTCCAAATGCAAGTG	GGCTTCATTTCCAGCTTCTTCA	Sybr
CAPN3	CCGCAACTTCCCAGACACTT	GGTCGTCGTCCTCCTCTAGGA	Sybr
MSTN	ACATCAGCAAAGATGCTATAAGACAAC	TCTGGACATCGTACTGATCAATCA	Sybr
CATHL5	ACCGCCTCCTGGAGCTAGAC	CTGTCCCCACACACTCTTTCAG	Sybr
CHST15	TGCATGCTGGATTATTCATTGC	TCACAGGCATGGCGTTGT	Sybr
TAC1	GCGACCAGATCAAGGAGGAA	GGGTCTCCGAGCGATTCTCT	Sybr
